# Expression of Translationally Controlled Tumor Protein in Human Kidney and in Renal Cell Carcinoma

**DOI:** 10.1155/2015/730390

**Published:** 2015-09-03

**Authors:** Maria R. Ambrosio, Bruno J. Rocca, Aurora Barone, Monica Onorati, Lucia Mundo, Filippo Crivelli, Franca Di Nuovo, Giulia De Falco, Maria T. del Vecchio, Sergio A. Tripodi, Piero Tosi

**Affiliations:** ^1^Section of Pathology, Department of Medical Biotechnology, University of Siena, Via delle Scotte 6, 53100 Siena, Italy; ^2^Section of Pathology, Ospedale di Circolo di Busto Arsizio, Presidio Ospedaliero di Saronno, Piazzale Borella 1, 21047 Saronno, Italy; ^3^Section of Pathology, Azienda Ospedaliera, “G. Salvini”, Viale C. Forlanini 121, 20024 Garbagnate Milanese, Italy; ^4^School of Biological and Chemical Sciences, Queen Mary University of London, Mile End Road, London E14NS, UK; ^5^Department of Medicine, Science and Neurosciences, University of Siena, Via delle Scotte 6, 53100 Siena, Italy; ^6^Section of Pathology, Azienda Ospedaliera Universitaria Senese, Viale Bracci 16, 53100 Siena, Italy

## Abstract

Translationally controlled tumor protein is a multifaceted protein involved in several physiological and biological functions. Its expression in normal kidney and in renal carcinomas, once corroborated by functional data, may add elements to elucidate renal physiology and carcinogenesis. In this study, translationally controlled tumor protein expression was evaluated by quantitative real time polymerase chain reaction and western blotting, and its localization was examined by immunohistochemistry on 84 nephrectomies for cancer. In normal kidney protein expression was found in the cytoplasm of proximal and distal tubular cells, in cells of the thick segment of the loop of Henle, and in urothelial cells of the pelvis. It was also detectable in cells of renal carcinoma with different pattern of localization (membranous and cytoplasmic) depending on tumor histotype. Our data may suggest an involvement of translationally controlled tumor protein in normal physiology and carcinogenesis. However, functional *in vitro* and *in vivo* studies are needed to verify this hypothesis.

## 1. Introduction

Translationally controlled tumor protein (TCTP) is a gene product ubiquitously expressed in all eukaryotes [1]. Its expression varies, depending on the cell type and developmental stage, with high level in mitotically active tissues and low level in resting cells [2]. In numerous experimental settings and biological systems, it has been demonstrated that TCTP expression is regulated by a wide range of extracellular signals and cellular conditions (growth factors, cytokines, proapoptotic/cytotoxic signals, and others), resulting in either upregulation or downregulation [3, 4]. Although the high degree of preservation and the abundance and the ubiquity of TCTP underline its critical role in the cell, the physiological functions of the protein are still poorly understood [1]. There is growing evidence that TCTP is a multifaceted protein associated with several different biological functions, such as development [5], cell cycle and division [6], cell proliferation and growth [7], cytoskeleton activity [8], chaperone-like activity [9], calcium binding [10], histamine release, and immune response [11]. In recent years attention has been focused on the possible role of TCTP in cancerogenesis [5]. Uncontrolled or promoted proliferation, loss of cell death and apoptosis, cell growth, and gene expression dysregulation are properties of tumor cells and all are influenced by TCTP activity [12–14]. Furthermore, TCTP has a crucial role in tumor reversion, a process by which some cancer cells lose their malignant phenotype [12]. TCTP is expressed in more than 500 tissues and cell types [15–17]. It has been found in whole kidney lysates of embryonic mouse [2] and in rat urinary organs [18]. In contrast, the protein has never been previously detected in human kidney and in renal cell carcinomas, as also reported by the Swiss-Prot proteomic bank (http://www.ebi.ac.uk/swissprot) [19]. For this reason, in a previous study performed to evaluate the expression of TCTP in prostate gland, human kidney tissue was used as negative control [20]. Surprisingly, we observed TCTP expression in normal renal structures.

Based on this preliminary data the present study is aimed at

- confirming TCTP expression on a larger series of samples of normal kidneys and evaluating its pattern of expression in the different compartments of the kidney,
- assessing TCTP expression in renal tumors.

## 2. Material and Methods

### 2.1. Patients

84 nephrectomies for cancer were collected at the Section of Pathology of Siena University Hospital (Siena, Italy). Preliminarily, neoplastic and nonneoplastic areas were selected from each sample and examined by frozen section procedure to confirm the presence or absence of the tumor. One neoplastic and one nonneoplastic area from each nephrectomy were snap-frozen in liquid nitrogen and stored at −80°C until being used for evaluation of TCTP expression protein level by western blotting (WB). The remaining of each specimen followed the standard procedure for histological and immunohistochemical analysis and were used for TCTP mRNA detection by quantitative real-time polymerase chain reaction (RT-qPCR) after microdissection.

### 2.2. Ethics Statement

Ethical approval for this study was obtained by the Institutional Review Board of the University of Siena (Italy). Informed written consent was obtained in all cases.

### 2.3. Histology

Representative samples of tumors and of normal renal parenchyma were taken, fixed in 10% buffered formalin, and embedded in paraffin. From each block, 4 *µ*m thick sections were cut and stained with haematoxylin and eosin. Tumor histotype, grade, and stage were established according to the last World Health Classification of tumors of urinary system and male genital organs [21] and to the updated International Society of Urologic Pathology (ISUP) grading system [22].

### 2.4. Detection of TCTP mRNA

Total RNA was extracted from cells isolated by microdissection from formalin-fixed, paraffin-embedded (FFPE) tissue blocks of normal (whole, medullary, and cortical regions) and both neoplastic benign and neoplastic malignant samples (oncocytoma, clear cell renal cell carcinoma, papillary carcinoma, chromophobe renal cell carcinoma, collecting duct carcinoma, and Wilm's tumor) using the RNA easy FFPE kit (Qiagen), following manufacturer's instructions. Reverse transcription was carried out using the QuantiTect Reverse Transcription Kit (Qiagen, CA). For each RNA specimen, a negative control was prepared by omitting the reverse transcriptase. The positive control was represented by placenta [20]. TCTP expression was analyzed in both normal and neoplastic samples by RT-qPCR, using TaqMan probes (Life Sciences, Applied Biosystems, CA) for TCTP and HPRT, used as housekeeping gene, according to the manufacturer's instructions. Relative quantification was calculated by the ΔΔCt method [23].

### 2.5. Western Blot Analysis

Frozen samples of renal tissue were reduced to small pieces with a razor blade and homogenized on ice three times (20 sec each) by a Polytron blender (Kinematica Lucerne, Switzerland) in lysis buffer (50 mM Tris-HCl, 5 mM magnesium acetate, 0.2% mM EDTA, 0.5 mM dithiothreitol, 10% (vol/vol) glycerol, and 0.2% (vol/vol) Triton X-100 (pH 7.5)) supplemented with a protease-inhibitor cocktail containing 4-(2-aminoethyl)benzenesulfonyl fluoride, pepstatin A, E64, bestatin, leupeptin, and aprotinin (Sigma Chemical Co.). Tissue homogenates were centrifuged at 750 ×g for 10 min at 4°C and the supernatant was assayed for total protein content and stored at −80°C. Extracts were separated by a 12% SDS-polyacrylamide gel (SDS-PAGE). After electrophoresis, proteins were transferred to nitrocellulose filters (Hybond-C, Amersham Biosciences Corp., Piscataway, NJ) for 1 h on ice in transfer buffer (20 mM Tris (pH 8.3), 0.15 M NaCl, and 0.1% Triton X-100). Filter was incubated in blocking solution for a specific site saturation for 1 h, followed by ON incubation with primary antibody (1 : 500), at 4°C. Filter was washed with TBST 6 × 5′, and then secondary antibody conjugated to HRP was incubated for 1 h at RT. After 30′ washing, detection was then obtained by chemiluminescence using the ECL kit (Amersham Biosciences Corp.), according to the manufacturer's instructions. The differences were normalized with an anti-actin antibody (1 : 1000). Densitometric analysis was carried out using the ImageMaster TotalLab software (Amersham Biosciences Corp.). The positive control was represented by placenta, as in other similar studies [20].

### 2.6. Immunohistochemistry

The most representative tumor blocks were selected on the basis of the morphological features. Immunohistochemical staining was performed on 4 ± 0.5 *μ*m thick sections of each block employing the Ultravision Detection System Antipolyvalent HRP (Ultra V Block) (LabVision, Fremont, CA, USA, Bio-Optica). All the procedures were carried out automatically by using the Bond-III machine. Slides were incubated with an anti-TCTP antibody (dilution: 1 : 25) and the reaction revealed using fuchsine (Dako, Milan, Italy) as chromogen. Sections were weakly counterstained with Harris' haematoxylin and examined under a light microscope. Nonimmune serum immunoglobulins were used as negative control, whereas the positive control was represented by placental tissue [20].

### 2.7. Staining Assessment

All of the samples were independently evaluated and scored by two investigators (Maria R. Ambrosio and Bruno J. Rocca), who were blinded to the clinicopathological information of the patients. TCTP protein expression levels were classified semiquantitatively combining the proportion and intensity of positively stained cells [24]. The percentage of positive-staining tumor cells was scored as follows: (1) <5% positive cells, (2) 5–50% positive cell, and (3) >50% positive cells. Staining intensity was scored as follows: (1) weak or not detectable staining, (2) moderate staining, and (3) strong staining [24]. Three different fields (at least 100 cells/field) were evaluated at ×200 magnification. In neoplastic samples TCTP protein expression level was evaluated and defined only in tumoural cells. The sum of the staining intensity score and the percentage score was used to define the TCTP protein expression level, low: 0–2; high: 3-4. The agreement between the two pathologists was about 90%. Cases with discrepancies were reviewed and discussed to reach the 100% of concordance.

### 2.8. Statistics

Statistical analysis was performed using a statistical software package (SigmaPlot 12.0, Systat Software), by employing Student's *t*-test, with *p* value <0.05 being considered significant.

## 3. Results

### 3.1. TCTP Is Present in Normal Kidney and in Renal Carcinomas

**Figure 1:**
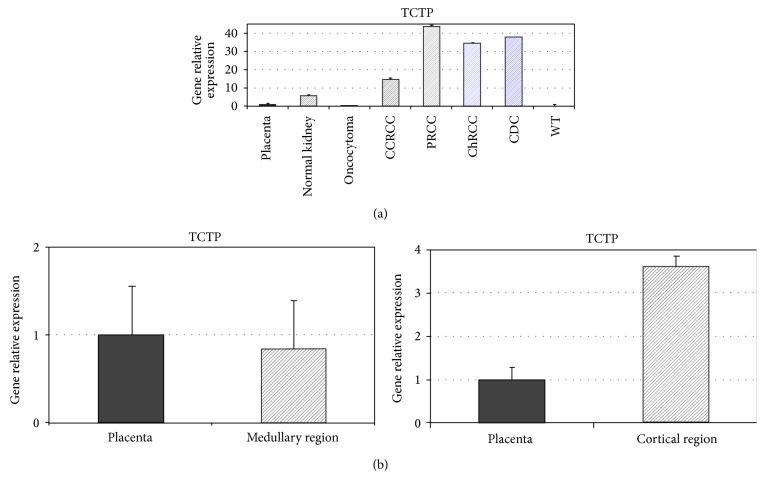
RT-qPCR analysis of TCTP in normal kidney and neoplastic specimens. Relative expression of TCTP mRNA was detected in normal kidney and in tumor samples. Values are expressed as the result of normalization using placenta samples. The graphs are representative of three different RT-qPCR experiments. Error bars represent standard deviation between duplicates. (a) TCTP mRNA was detected in normal kidney and, at higher level, in malignant tumor samples. (b) In normal kidney, a higher TCTP mRNA level was detected in the cortical region with respect to medullary region (CCRCC: clear cell renal cell carcinoma; PRCC: papillary renal cell carcinoma; ChRCC: chromophobe renal cell carcinoma; CDC: collecting duct carcinoma; WT: Wilm's tumour).

**Figure 2:**
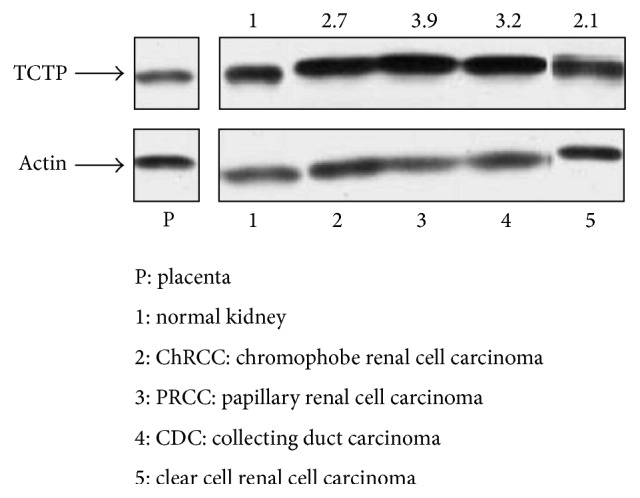
Western blotting analysis of TCTP protein expression in normal kidney and neoplastic specimens. WB was performed to confirm TCTP protein expression in normal kidney and in different renal tumors. A specific band of the MW of 22 kDa was detected in all the neoplastic and nonneoplastic specimens. Placenta was used as positive control. Relative expression of TCTP was measured with respect to actin, used as control. WB was carried out in triplicate. Densitometric analysis revealed higher expression of TCTP in tumor samples, with respect to normal kidney. (1) Normal kidney; ((2)–(5)) tumor samples: (2) ChRCC; (3) PRCC; (4) CDC; (5) CCRCC (WB: western blotting; MW: molecular weight; ChRCC: chromophobe renal cell carcinoma; PRCC: papillary renal cell carcinoma; CDC: collecting duct carcinoma; CCRCC: clear cell renal cell carcinoma).

To evaluate the expression of TCTP, total RNA extracted from normal and neoplastic tissues was examined by RT-qPCR. We detected its expression at the mRNA level in normal kidney, being significantly higher in the cortical region (*p* < 0.05, *p* = 0.01) (Figures 1(a) and 1(b)). We then analyzed its expression level in neoplastic samples of renal tumors, representative of different histotypes. Relative quantification indicated that the expression of TCTP is significantly higher in renal cell cancers than in normal tissue and benign tumors (oncocytoma) (*p* < 0.05, *p* = 0.03); Wilm's tumors showed no TCTP expression (Figure 1(a)). TCTP protein expression was then confirmed in the specimens by western blotting. A specific band of the approximate molecular weight of 22 kDa was detected in all the neoplastic and nonneoplastic specimens (Figure 2). Higher expression of the protein was detected in tumor tissues, confirming the RT-qPCR results (Figure 2).

### 3.2. TCTP Staining Is Different in the Different Sites of Human Kidney and in the Different Tumor Histotypes

**Figure 3:**
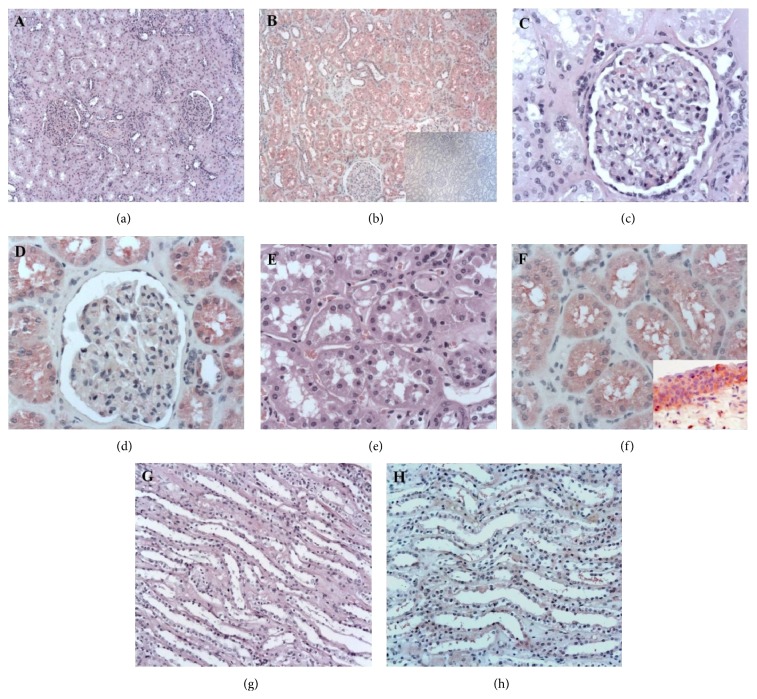
Immunohistochemical evaluation in normal kidney region. At low power, TCTP staining is present in all the structures belonging to the cortical region ((a) haematoxylin and eosin (H&E); (b) TCTP staining; ((a)-(b)) ×50; (b) inset, negative control, ×50); at higher power, weak TCTP expression is observed in glomeruli ((c) H&E; (d) TCTP staining; ((c)-(d)) ×200) and strong positivity is shown by proximal tubules ((e) H&E; (f) TCTP staining; ((e)-(f)) ×200). In the medullary region, TCTP immunoreactivity was very low or absent ((g) H&E; (h) TCTP staining, ×200). TCTP expression is also present in the transitional epithelium of the renal pelvis ((f) inset, TCTP staining, ×200).

The different pattern of expression of TCTP was evaluated by immunohistochemistry in all the 84 FFPE nephrectomies. For each surgical specimen the most representative neoplastic and nonneoplastic area were selected. In normal tissue TCTP was found within the kidney cortex (both glomeruli and tubules) (Figures 3(a) and 3(b)). In glomeruli, a low level of TCTP expression was detectable in podocytes with variations from cell to cell and in endothelial and mesangial cells (Figures 3(c) and 3(d)). The protein expression was higher in the proximal tubules and lower in the distal ones (Figures 3(e) and 3(f)). In the medulla, TCTP immunoreactivity was very low or absent and present only in the thick segment of Henle's loop (Figures 3(g) and 3(h)). TCTP expression was also observed in the transitional epithelium of the renal pelvis (Figure 3(f), inset), being mostly concentrated in cells of the basal layer. In all these compartments TCTP expression was cytoplasmic. The negative control is shown in the inset of Figure 3(b).

**Figure 4:**
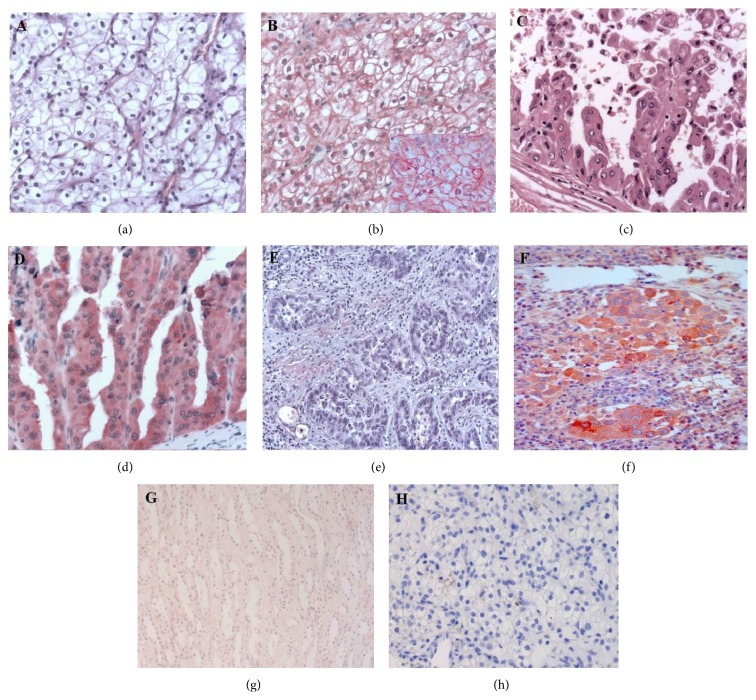
Immunohistochemical evaluation in cancer specimens. In clear cell carcinoma a membrane positivity is observed ((a) H&E; (b), (b) inset, TCTP staining; ((a)-(b)) ×200; (b) inset ×400); in papillary carcinoma ((c) H&E; (d) TCTP staining; ((c)-(d)) ×100) and in collecting duct carcinoma, the staining is cytoplasmic ((e) H&E; (f) TCTP staining; ((e)-(f)) ×200). The comparison with nonneoplastic tissue from the same samples is shown ((g) TCTP stain, ×200) as well as the negative control ((h) ×200).

Among the tumoural specimens, 4 were oncocytomas, 36 were clear cell carcinoma, 25 were papillary carcinoma, 12 were chromophobe carcinoma, 4 were collecting duct carcinoma, and 3 were Wilm's tumors. All the specimens, with the exception of oncocytomas and Wilm's tumors, expressed TCTP. The immunoreactivity of neoplastic cells was always intense; in clear cell carcinoma it was membranous (Figures 4(a) and 4(b), inset), whereas it was cytoplasmatic in papillary carcinoma (Figures 4(c) and 4(d)), in collecting duct carcinoma (Figures 4(e) and 4(f)), and in chromophobe carcinoma. The comparison with a nonneoplastic region of the same sites is demonstrated in Figure 4(g), whereas the negative control is shown in Figure 4(h). No differences were found among the various grades and stages.

The results on the expression of TCTP in both nonneoplastic and neoplastic samples are summarized in Table 1.

## 4. Discussion

TCTP is a protein already known to be expressed in almost all the human organs but not in the human kidney ([2, 6, 19], http://www.ebi.ac.uk/swissprot). To the best of our knowledge, only four previous studies addressing TCTP expression in the kidney have been published so far; however, they were performed on rat and mouse and mainly focused on TCTP content in cell lysates of the whole organ [2, 4, 18, 25]. All these studies suggested a potential role of TCTP in renal physiology. In our study we demonstrate for the first time the expression of the protein in human kidney. We detect that TCTP is mainly expressed in the cortex and in particular in the cytoplasm of the proximal tubular cells, opening to the possibility to link this expression to urine formation, as suggested by Sheverdin et al. [18], and, for instance, to calcium absorption, the proximal tubule being one of the major sites of calcium absorption. Our findings seem to be in contrast to what has been previously reported in the literature and in proteomic banks, that is, the absence of the protein in human kidney. We hypothesize that the lack of expression in detecting the protein might be related to a sampling error for which TCTP mRNA and protein were analyzed only in medullary region, in which the protein is exactly not present. However, functional data should be collected to try to validate this hypothesis. Many cellular processes which are regulated by TCTP (cell proliferation, cell death, apoptosis, stress and heat shock response, gene regulation, immune system activity, and pluripotency) could, if dysregulated, lead to cancer [5–11]. It has been found that TCTP expression increases in some types of cancer (colon, lung, prostate, breast, and melanoma) and decreases after tumor reversion, as demonstrated in different cell lines and by knockdown experiments that showed a suppression of the malignant phenotype following inhibition of TCTP expression [12, 13]. A large-scale screening analysis of genes expressed by parental tumor cells in comparison with revertants showed that TCTP had the most striking different expression, significantly higher in neoplastic cells than in nonneoplastic ones [12]. Two studies confirmed these findings in several different tumor cell lines and demonstrated that the mechanism of neoplastic reversion determined by TCTP relies on a reorganization of the process of cell cycle arrest, apoptosis, and terminal differentiation as a form of rerouting and trigger of the whole machinery that enables the tumor cells to quit the malignant pathway [12, 13]. Such a system could override the genetic changes in cancer not necessarily correcting the genetic alterations but instead bypassing them and reprogramming the cancer cells to recover some of their normal functions. In the present study, we describe the expression of TCTP in clear cell, chromophobe, papillary, and collecting duct carcinomas which is much higher than in normal tissue and benign tumors (i.e., oncocytoma). The negativity for TCTP in Wilm's tumor may be related to the different histogenesis of the neoplasm (i.e., abnormally persistent metanephric blastemal cells). We also observed a different pattern of localization of TCTP in the various tumor histotypes: membranous in clear cell carcinomas, cytoplasmatic in papillary, chromophobe, and collecting duct carcinoma. Membranous expression of TCTP has never been described so far [26] and hence it is possible to hypothesize that the membranous localization of the protein in clear cell carcinoma may be related to the content of the cytoplasm of the neoplastic cells (i.e., abundant glycogen and lipid) that may displace the TCTP towards the plasma membrane. No significant differences of expression were found in the various histological grades and stages of the carcinomas.

Both the hypothetical involvement in renal physiology and the eventual implication of TCTP in renal carcinogenesis are at moment only speculative, since functional* in vitro* and* in vivo* data are still lacking. Moreover, it is known that protein expression in neoplasia can also be an effect of transformation rather than a cause. A study is currently ongoing in our laboratory to verify the hypotheses arising from the present data. Whether confirmed, since recent studies have provided the preclinical proof of principle of the therapeutic role of an antisense oligonucleotide-mediated TCTP knockdown in those cancers in which the protein is expressed [27], TCTP might be considered in the setting of a more tailored cancer therapy.

## 5. Conclusions

The findings of this study let us hypothesize that TCTP is involved in renal tubular functions, since tubular cells express the protein at the highest level, and that it might be implicated in renal carcinogenesis since it is expressed in all the histotypes of renal cell carcinoma and not in benign tumors. However, functional* in vivo* and* in vitro* studies are needed to confirm these hypotheses.

## Figures and Tables

**Table 1:** Immunohistochemical results in normal and neoplastic kidney samples. The staining for TCTP is listed, with higher protein expression in cortical regional and malignant tumours.

Cell tissue	*N*	TCTP protein expression		Pattern of expression
		High	Low/absent	
Cortical region	84	80	4	Cytoplasmatic
Proximal tubules				Strong
Glomeruli				Weak
Medullary region	84	5	79	Cytoplasmatic
Clear cell carcinoma	36	34	2	Membranous
Papillary carcinoma	25	24	1	Cytoplasmatic
Chromophobe carcinoma	12	11	1	Cytoplasmatic
Collecting duct carcinoma	4	4	0	Cytoplasmatic
Wilm's tumors	3	0	3	nd
Oncocytomas	4	0	4	nd

*N*: number of cases; nd: not detectable.
